# Supplementary material for: Larval settlement and metamorphosis in a marine gastropod in response to multiple conspecific cues
Source: PeerJ. 2016 Jul 28;4:e2295. doi: 10.7717/peerj.2295 (PMC4974925; doi:10.7717/peerj.2295)
Supplement: Table S1 [file peerj-04-2295-s001.docx]

**Table A1. Settlement data as analyzed using proportions.** Analyses of variance conducted on the proportion of larvae settled at each of four time points (12, 24, 36, and 48 hours). Analyses conducted on arcsine square root-transformed data due to failure to meet the assumption of normality. p-values significant at < 0.05 are highlighted in bold.

*12 hours:*

| Source of Variation | df | SS | MS | F | p |
| --- | --- | --- | --- | --- | --- |
| Block Effect | 2 | 3808.75 | 1904.38 | ---- | **----** |
| Adult Conditioned Water (ACW) | 1 | 103.10 | 103.10 | 0.69 | 0.408 |
| Pedal Mucus Glass (PMG) | 1 | 0.96 | 0.96 | 0.01 | 0.936 |
| Potassium Chloride (KCl) | 1 | 5647.01 | 5647.01 | 37.98 | **< 0.001** |
| PMG*ACW | 1 | 15.25 | 15.25 | 0.10 | 0.750 |
| PMG*KCl | 1 | 151.44 | 151.44 | 1.02 | 0.317 |
| ACW*KCl | 1 | 2318.35 | 2318.35 | 15.59 | **< 0.001** |
| ACW*PMG*KCl | 1 | 241.34 | 241.34 | 1.62 | 0.207 |
| Error | 62 | 9218.95 | 148.69 | 0.00 |  |
| Total | 71 | 21802.54 |  |  |  |

*24 hours:*

| Source of Variation | df | SS | MS | F | p |
| --- | --- | --- | --- | --- | --- |
| Block Effect | 2 | 3561.12 | 1780.56 | ---- | ---- |
| Adult Conditioned Water (ACW) | 1 | 184.61 | 184.61 | 1.09 | 0.301 |
| Pedal Mucus Glass (PMG) | 1 | 0.70 | 0.70 | 0.00 | 0.949 |
| Potassium Chloride (KCl) | 1 | 8249.06 | 8249.06 | 48.69 | **< 0.001** |
| PMG*ACW | 1 | 257.00 | 257.00 | 1.52 | 0.223 |
| PMG*KCl | 1 | 47.52 | 47.52 | 0.28 | 0.598 |
| ACW*KCl | 1 | 2176.13 | 2176.13 | 12.84 | **< 0. 001** |
| ACW*PMG*KCl | 1 | 308.72 | 308.72 | 1.82 | 0.182 |
| Error | 62 | 10504.68 | 169.43 | 0.00 |  |
| Total | 71 | 25628.40 |  |  |  |

*36 hours:*

| Source of Variation | df | SS | MS | F | p |
| --- | --- | --- | --- | --- | --- |
| Block Effect | 2 | 3690.82 | 1845.41 | ---- | ---- |
| Adult Conditioned Water (ACW) | 1 | 1296.93 | 1296.93 | 10.11 | **0.002** |
| Pedal Mucus Glass (PMG) | 1 | 506.36 | 506.36 | 3.95 | 0.0514 |
| Potassium Chloride (KCl) | 1 | 4041.01 | 4041.01 | 31.51 | **< 0.001** |
| PMG*ACW | 1 | 532.14 | 532.14 | 4.15 | **0.046** |
| PMG*KCl | 1 | 1043.33 | 1043.33 | 8.13 | **0.006** |
| ACW*KCl | 1 | 3332.55 | 3332.55 | 25.98 | **< 0.001** |
| ACW*PMG*KCl | 1 | 280.06 | 280.06 | 2.18 | 0.145 |
| Error | 62 | 7952.36 | 128.26 | 0.00 |  |
| Total | 71 | 22932.08 |  |  |  |

*48 hours:*

| Source of Variation | df | SS | MS | F | p |
| --- | --- | --- | --- | --- | --- |
| Block Effect | 2 | 2981.98 | 1490.99 | ---- | ---- |
| Adult Conditioned Water (ACW) | 1 | 1167.98 | 1167.98 | 8.21 | **0.006** |
| Pedal Mucus Glass (PMG) | 1 | 531.98 | 531.98 | 3.74 | **0.058** |
| Potassium Chloride (KCl) | 1 | 4877.59 | 4877.59 | 34.29 | **< 0.001** |
| PMG*ACW | 1 | 737.22 | 737.22 | 5.18 | **0.026** |
| PMG*KCl | 1 | 1255.76 | 1255.76 | 8.83 | **0.004** |
| ACW*KCl | 1 | 3121.16 | 3121.16 | 21.94 | **< 0.001** |
| ACW*PMG*KCl | 1 | 407.50 | 407.50 | 2.87 | 0.096 |
| Error | 62 | 8818.34 | 142.23 | 0.00 |  |
| Total | 71 | 24183.96 |  |  |  |
