# Supplementary material for: Larval settlement and metamorphosis in a marine gastropod in response to multiple conspecific cues
Source: PeerJ. 2016 Jul 28;4:e2295. doi: 10.7717/peerj.2295 (PMC4974925; doi:10.7717/peerj.2295)
Supplement: Figure S1 [file peerj-04-2295-s002.docx]

**Fig. A1. Optimization of experimental parameters.** A discussion of the results of each graph is provided. Each graph represents the cumulative proportion of larvae settled through time under different trial conditions. All values are treatment totals out of a sample size of 30 larvae (10 larvae per glass; three glasses). These graphs were visually analyzed to determine the optimal value of each parameter; no statistics were conducted. Only the variable being tested was altered in each trial; the optimal value was used for all other variables as described in Table 1 of the main text.


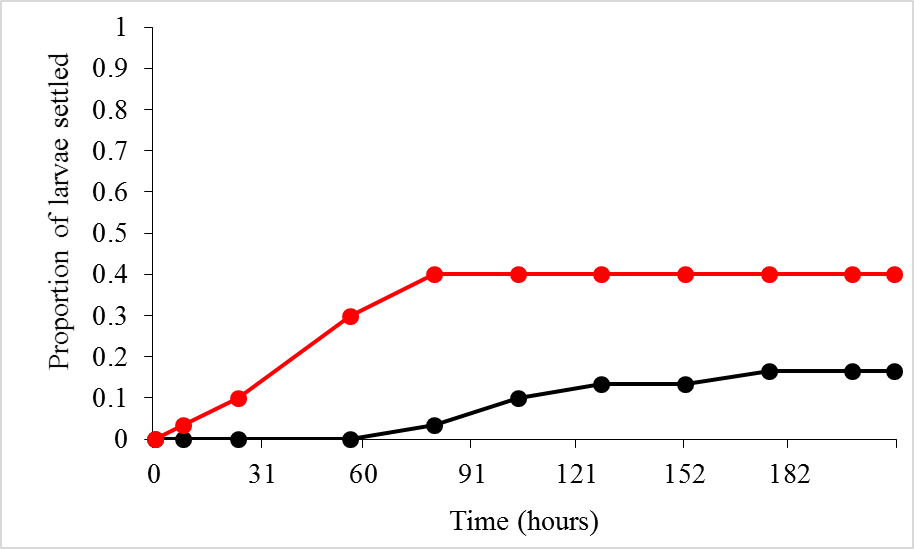


**Fig. A1a. Trial duration.** Data testing ACW (red line) against FSW (black line). By 72 hours, mortality overtakes settlement in the ACW treatment (mortality not shown), and spontaneous settlement begins to increase in the FSW treatment.


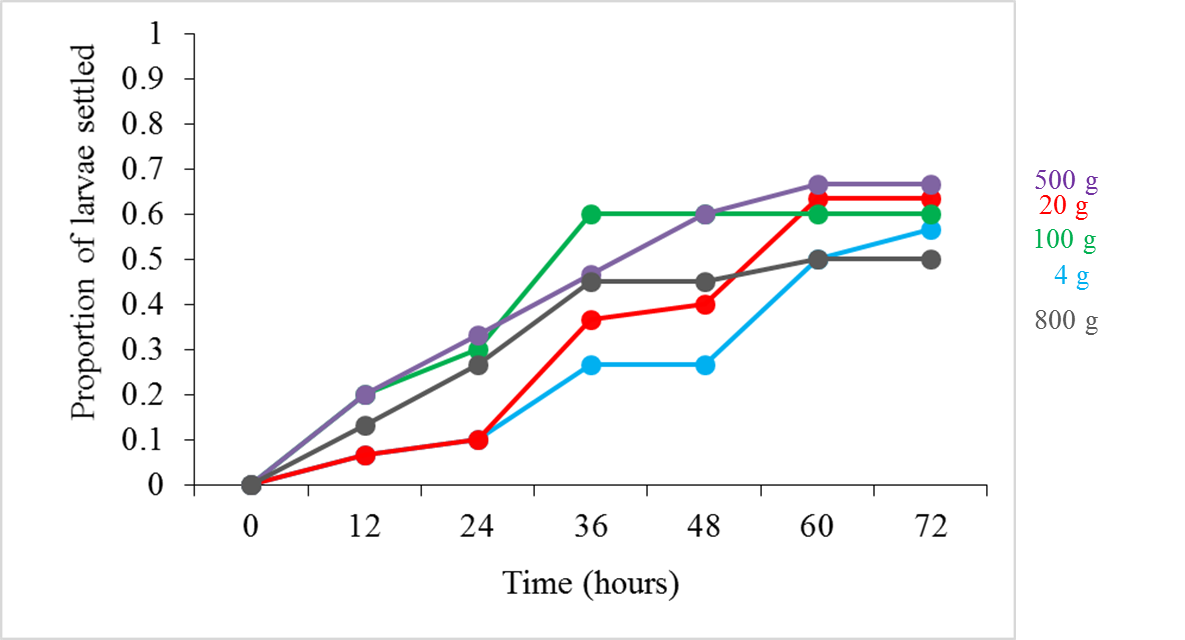


**Fig. A1b. Adult mass.** Proportion of larvae settled during a run where all ACW was prepared for 12 hours while varying adult mass (4g, blue line; 20g, red; 100g, green; 500g, purple; or 800g, gray, of adult mass per liter of FSW). The treatment made with 800 g of adult mass showed high mortality by the end of the experiment (not shown). 100 g and 500 g were comparable, but 100 g was chosen because it requires fewer adults for preparation.


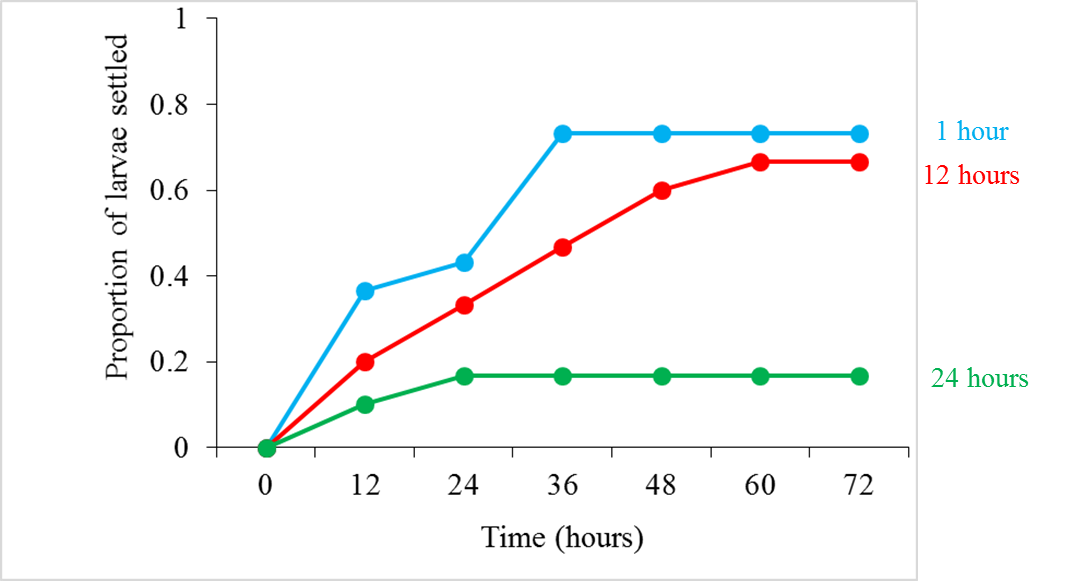


**Fig. A1c. Cue preparation time.**  Data from a run where all ACW was prepared for 1 (blue line), 12 (red), or 24 hours (green) with 500 g adult mass per liter of FSW. ACW prepared for 24 hours resulted in total larval mortality by 48 h during the settlement trial. Although 1 hour and 12 hour preps were roughly comparable, 12 hours of preparation time was chosen for logistical reasons.


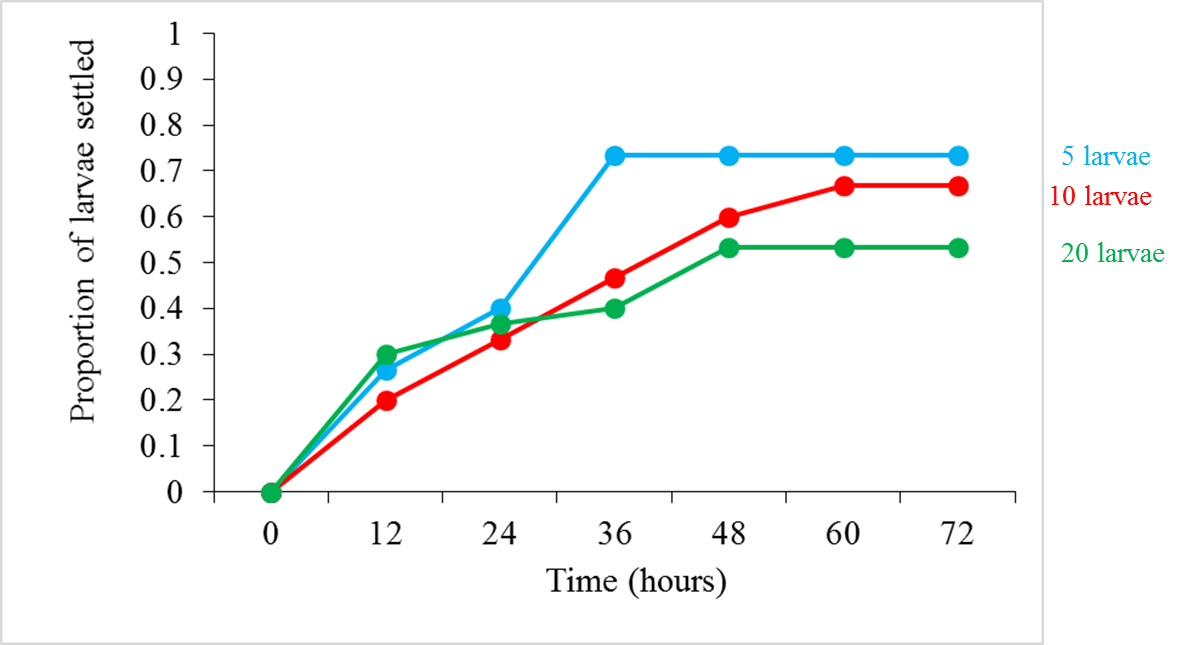


**Fig. A1d. Larvae per glass.** Proportion of larvae settled during a run of 5 (blue line), 10 (red), or 20 larvae (green) in each 20 ml glass of ACW. High mortality was seen with 20 larvae per glass (not shown), and individual larvae were difficult to examine at that density. The optimal value chosen for the experiment was 10 larvae per glass.


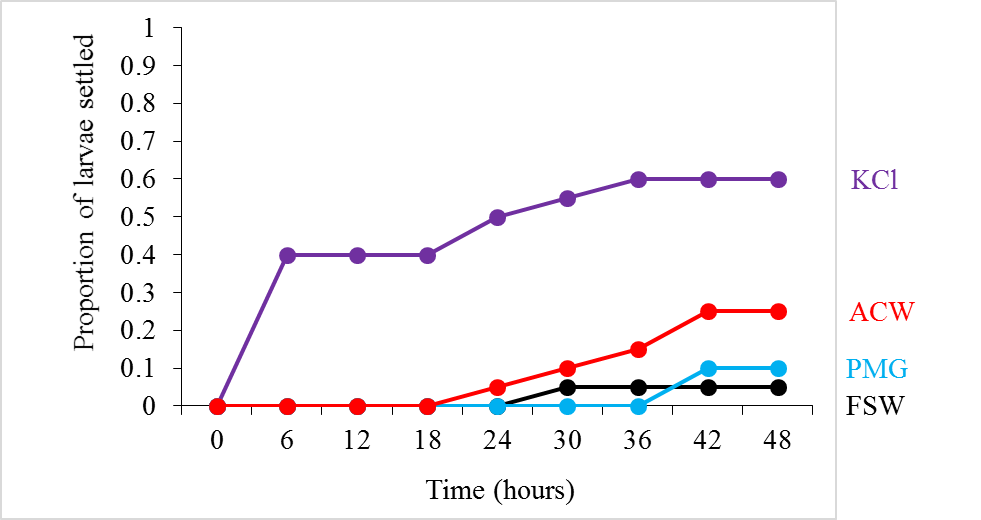


**Fig. A1e. Time interval.** Proportion of larvae settled from a run of ACW (red line), PMG (blue), and KCl (purple; i.e., all main settlement factors) where data were collected every six hours. Data was ultimately taken every 12 hours because settlement did not change much at six-hour intervals.
